# A refractory human T-cell leukemia virus type 1-associated myelopathy/tropical spastic paraparesis patient with lymphoma-type adult T-cell leukemia/lymphoma: A case report and review of the literature

**DOI:** 10.1097/MD.0000000000027450

**Published:** 2021-10-08

**Authors:** Keiko Tamaki, Hidekazu Mera, Sho Takeshita, Shinsuke Fujioka, Maki Goto, Taichi Matsumoto, Yoshihisa Yamano, Yasushi Takamatsu, Yoshio Tsuboi

**Affiliations:** a Department of Neurology, Fukuoka University, Japan; b Division of Medical Oncology, Hematology and Infectious Diseases, Fukuoka University, Japan; c Department of Drug Informatics and Translational Research, Faculty of Pharmaceutical Sciences, Fukuoka University, Japan; d Division of Neurology, St. Marianna University School of Medicine, Japan.

**Keywords:** adult T-cell leukemia/lymphoma (ATL), central infiltration, clonality analysis, HTLV-1-associated myelopathy/tropical spastic paraparesis (HAM/TSP)

## Abstract

**Rationale:**

Adult T-cell leukemia/lymphoma (ATL) and human T-cell leukemia virus type 1 (HTLV-1)-associated myelopathy/tropical spastic paraparesis (HAM/TSP) are caused by HTLV-1, but the coexistence of both disorders is rare. The estimated incidence is approximately 3%.

**Patient concerns:**

A 54-year-old man was unable to stand up because of spastic paraparesis 1 month after the onset. He developed lymphadenopathy in the left supraclavicular fossa 5 months after the onset. The spastic paraplegia and sensory symptoms below the thoracic spinal cord level worsened.

**Diagnoses:**

Both blood and cerebrospinal fluid (CSF) tests were positive for anti-HTLV-1 antibodies. The patient was diagnosed with rapidly progressive HAM/TSP. He was also diagnosed with lymphoma-type ATL by the biopsy specimen of the lymph node. CSF examination at the time of symptom exacerbation showed abnormal lymphocytes, suggesting central infiltration of the ATL in the central nervous system.

**Interventions:**

Methylprednisolone pulse therapy and oral prednisolone maintenance therapy were administered for rapidly progressive HAM/TSP. Intrathecal injection of methotrexate was administered for the suggested central infiltration of the ATL.

**Outcomes:**

Methylprednisolone pulse therapy and intrathecal injection of methotrexate did not improve the patient's exacerbated symptoms. Five months later, clumsiness and mild muscle weakness of the fingers appeared, and magnetic resonance imaging showed swelling of the cervical spinal cord. Clonality analysis showed monoclonal proliferation only in the DNA of a lymph node lesion, but not in the CSF and peripheral blood cells.

**Lessons:**

This was a case of rapidly progressive HAM/TSP associated with lymphoma-type ATL that was refractory to steroids and chemotherapy. The pathogenesis was presumed to involve ATL cells in the brain and spinal cord because of the presence of abnormal lymphocytes in the CSF, but DNA analysis could not prove direct invasion. This case suggests that when we encounter cases with refractory HAM/TSP, it should be needed to suspect the presence of ATL in the background.

## 1 Introduction

Epidemiologically, human T-cell leukemia virus type 1 (HTLV-1) infects people predominantly in Japan, sub-Saharan Africa, South America, the Caribbean area, Middle East, and Australo-Melanesia and HTLV-1 infection is estimated to affect at least 5 to 10 million people worldwide.^[1]^ The prevalence in Japan is estimated to be at least 1.08 million people according to a 2006 to 2007 survey.^[2]^ The main transmission routes are vertical from mother to child via breastfeeding and horizontally through sexual intercourse. Although the majority of HTLV-1-infected individuals remain asymptomatic for life, approximately 0.25% to 3% of HTLV-1-infected individuals develop HTLV-1-associated myelopathy/tropical spastic paraparesis (HAM/TSP),^[3]^ and they develop adult T-cell leukemia/lymphoma (ATL) in 3% to 5% of cases.^[4]^ The average age of onset of HAM/TSP is 43.8 years, and HAM/TSP is more common in women, with a male:female ratio of 1:2 to 3.^[5]^ The age of onset of ATL is biased toward the elderly, peaking in the late 60 s, predominantly affecting men, with a male:female ratio of 1.2:1.^[4]^ ATL is classified into 4 subtypes: smoldering, chronic, lymphoma, and acute. In lymphoma-type ATL, abnormal lymphocytes in the peripheral blood account for less than 1%.^[6]^ The development of ATL in HAM/TSP patients has been considered rare, but the latest survey found that the rate was 3%, which is higher than previously thought.^[7]^

A patient was diagnosed with rapidly progressive HAM/TSP, in which lymph node swelling appeared during the course of the illness and was diagnosed with lymphoma-type ATL. Steroid therapy for rapidly progressive HAM/TSP and chemotherapy for ATL were ineffective for myelopathy, and the possibility of central infiltration of ATL was suspected. This case is reported along with a review of the literature.

## 2 Case report

A 54-year-old man had difficulty walking due to weakness and numbness in his lower limbs in January 2019, and was unable to stand up at the end of January. He was referred to the Department of Neurology at a local hospital. Neurological findings included spastic paraparesis and mild bladder and rectal dysfunction. Both blood and cerebrospinal fluid (CSF) tests were positive for anti-HTLV-1 antibodies. Anti-aquaporin-4 antibody was negative. The patient was diagnosed with rapidly progressive HAM/TSP according to the established diagnostic criteria.^[8]^ The diagnosis of rapidly progressive HAM/TSP was made by progressing to a Osame motor disability score grade 5 or higher within 2 years of the onset of motor symptoms (Table 1).^[8,9]^ Methylprednisolone (mPSL) pulse therapy (1000 mg/day for 3 days) was administered for a total of 3 cycles. He was referred to our department in February because of a poor response to steroid treatment. His father was from Fukuoka and his mother was from Nagasaki; both prefectures are located in the Kyushu area of Japan, where the frequency of HTLV-1 carriers is relatively high. He had no family history of HAM/TSP or ATL. On admission, neurological examination showed spastic paraparesis and hyperreflexia of the lower limbs with Babinski signs. The muscle strength by manual muscle testing of his upper limbs was normal, but the iliopsoas and hamstrings decreased to 3/5 and the tibialis anterior decreased to 4/5 on the right. Sensory dysfunction was noted below the 12^th^ thoracic spinal level, and bladder and rectal dysfunctions were noted. Treatment with oral prednisolone (PSL) 25 mg/day and rehabilitation was initiated. The PSL dose was gradually decreased, and in May, when the dose reached 7.5 mg/day, the spasticity of the lower limbs worsened.

**Table 1:** Osame motor disability score.

Grade	Motor disability
0	No walking or running abnormalities
1	Normal gait but runs slowly
2	Abnormal gait (stumbling, stiffness)
3	Unable to run
4	Needs handrail to climb stairs
5	Needs a cane (unilateral support) to walk
6	Needs bilateral support to walk
7	Can walk 5 to 10 m with bilateral support
8	Can walk 1 to 5 m with bilateral support
9	Cannot walk, but able to crawl
10	Cannot crawl, but able to move using arms
11	Cannot move around, but able to turn over in bed
12	Cannot turn over in bed
13	Cannot even move toes

**Figure 1:**
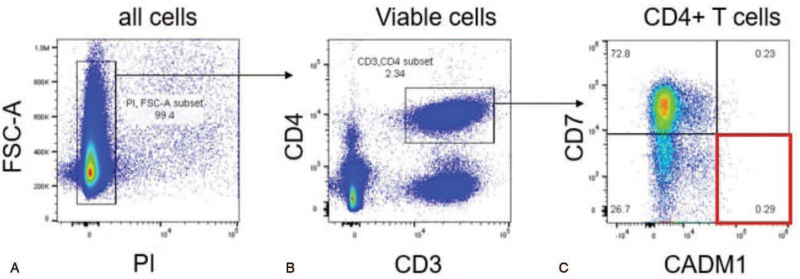
Flow cytometric analysis for ATL cells in CSF viable cells (PI−) were selected first (A). Viable cells include 2.34% CD4+ T cells (CD3+, CD4+ fraction) (B). CD4+ T cells include 0.29% ATL cells (CADM1+CD7− cells). This result is not the profile of the ATL. ATL = adult T-cell leukemia/lymphoma, CSF = cerebrospinal fluid.

The patient was readmitted to our hospital in June. Treatment with mPSL pulse therapy was administered without neurological improvement. CSF biomarker levels before mPSL pulse therapy were neopterin 5 pmol/mL and C-X-C motif chemokine 10 (CXCL10) 977.1 pg/mL, indicating that the disease activity of HAM/TSP was low to moderate. In mid-June, the patient developed lymphadenopathy in the left supraclavicular fossa. The biopsy specimen showed diffuse type lymphoma (CD3+, CD4+, CD5+, CD7−, CD8−, CD25+), and he was diagnosed with lymphoma-type ATL. In the latter half of July, there was a total loss of motor function of the lower limbs, with complete sensory disorders below the 8^th^ thoracic spinal cord, and dysuria became prominent. No abnormal findings were observed on contrast-enhanced cervical-thoracic spine magnetic resonance imaging (MRI). Cell analysis of CSF showed 9% atypical lymphocytes and a small number of abnormal lymphocytes, and central involvement of ATL cells was suspected. Flow cytometric analysis of CSF cells showed that the percentage of ATL cells (CADM1+ CD7-cells) was only 0.29% in CD4+ T cells,^[10]^ which was not the profile of ATL (Fig. 1). However, since exacerbation of neurological symptoms due to central involvement of ATL was suspected, intrathecal injection of methotrexate 15 mg with hydrocortisone 50 mg was administered once a week for a total of 4 times. The atypical and abnormal lymphocytes in the CSF disappeared, but no neurological improvement was observed. Contrast-enhanced thoracic lumbar spine MRI at the end of August showed a very faint hyperintensity region on T2-weighted imaging (T2WI) in part of the upper to middle thoracic spinal cord (both lateral cords to part of the posterior cord).

**Figure 2:**
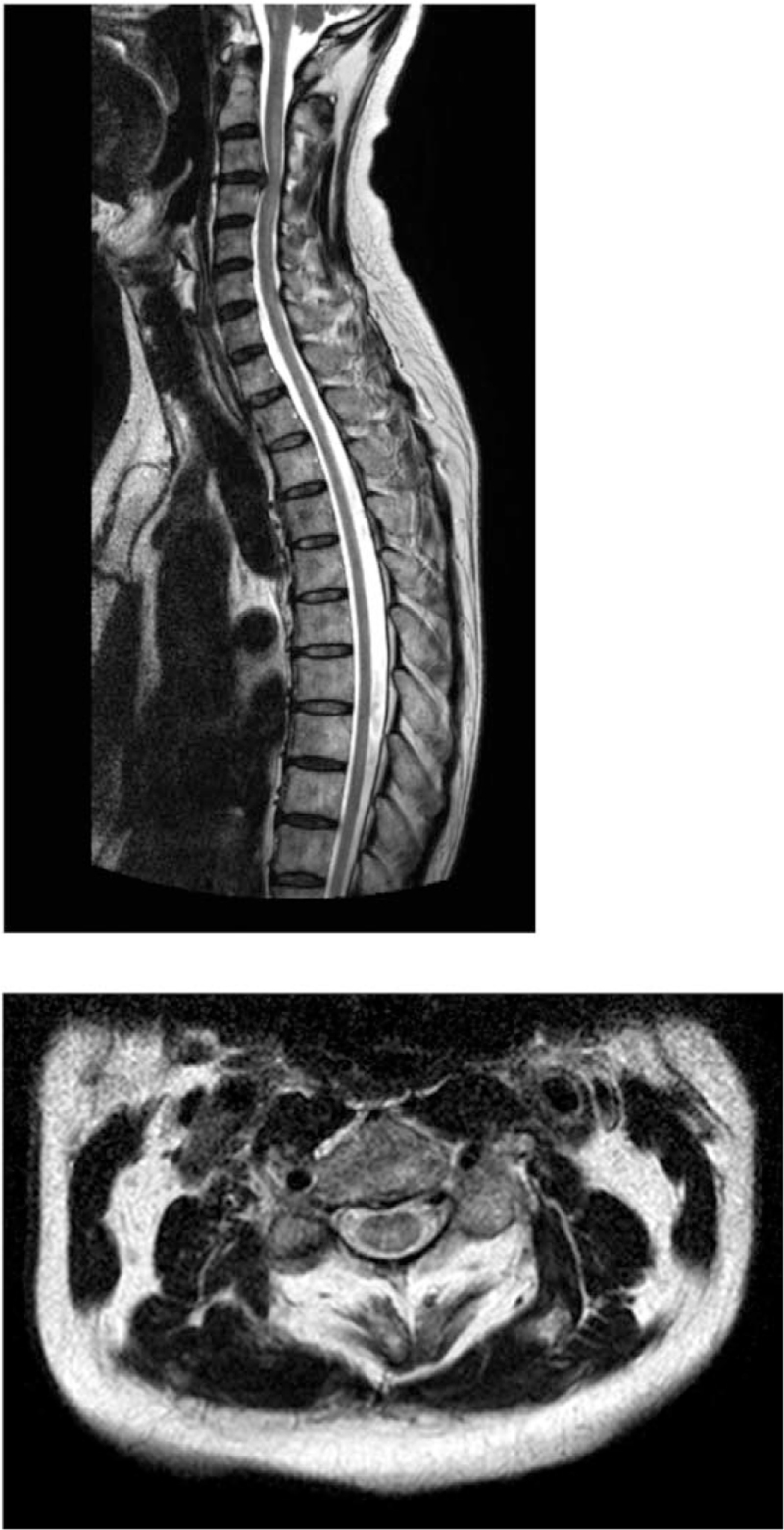
Contrast-enhanced cervical thoracic spine MRI A very faint hyperintensity is suspected on T2WI from the cervical spinal cord to a part of the middle thoracic spinal cord (both lateral cords to part of the posterior cord). Compared to MRI 6 months earlier, this finding is more evident in the cervical cord. No abnormal contrast findings were observed. MRI = magnetic resonance imaging, T2WI = T2-weighted imaging.

**Figure 3:**
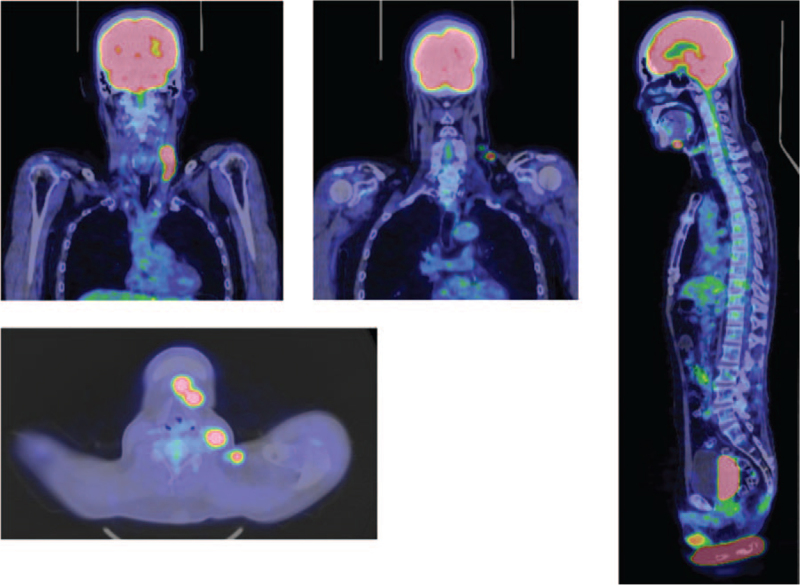
PET-CT In the regions of the bilateral accessory nerves, left lower inner deep neck, left submandibular, and bilateral supraclavicular fossae, lymphadenopathy, and abnormal FDG accumulation were observed. The left supraclavicular lymphoma lesion had shrunk compared to the lesion 8 months earlier, but multiple lymphoma lesions newly appeared. In addition, there is an increase in accumulation in the cervical spinal cord, and there is a region where the increase is particularly strong at the C4 level. These findings suggest the presence of cervical cord infiltration. There is also localized abnormal accumulation in the lower thoracic spinal cord, which might have infiltrated. FDG = fluorodeoxyglucose, HTLV-1 = human T-cell leukemia virus type 1, PET–CT = positron emission tomography–computed tomography.

**Figure 4:**
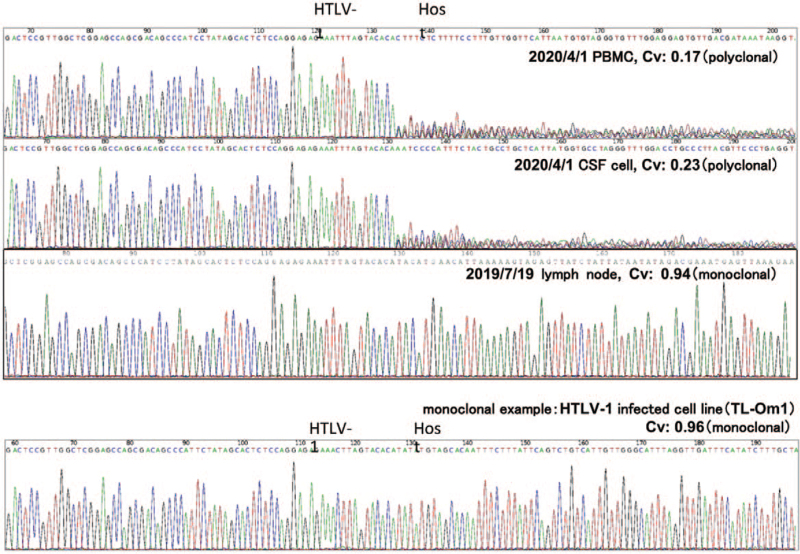
Clonality analysis of HTLV-1 infected cells. The Cvs of PBMC, CSF cells, lymph node, and monoclonal HTLV-1 infected cell line (TL-Om1) are shown. The Cvs of PBMC and CSF cells were low, whereas the Cvs of the lymph node were high (0.94), suggesting that ATL cells could not be detected in PBMC and CSF cells, whereas ATL cells were dominant in the lymph node. ATL = adult T-cell leukemia/lymphoma, CSF = cerebrospinal fluid, Cvs = clonality values, HTLV-1 = human T-cell leukemia virus type 1, PBMC = peripheral blood mononuclear cell.

The patient did not consent to systemic chemotherapy, and he was transferred to a rehabilitation hospital in October. In March 2020, a weakness in the left hand appeared. Neurological findings showed pseudoathetosis in both fingers, and the thumb localizing test^[11]^ showed bilateral position sense impairment, which was more pronounced on the right. The upper limb tendon reflexes were mildly exaggerated. Contrast-enhanced cervical thoracic spine MRI showed a very faint hyperintensity on T2WI from the cervical cord to a part of the middle thoracic cord (both lateral cords to part of the posterior cord) (Fig. 2), but no enhancement was observed. In addition, positron emission tomography-computed tomography showed abnormal fluorodeoxyglucose accumulation in multiple cervical lymph nodes and the cervical and thoracic spinal cord, suggesting spinal cord infiltration (Fig. 3). Head MRI showed a wide range of hyperintensities in the subcortical white matter on T2WI/fluid attenuated inversion recovery, and small hyperintensity in the left middle cerebellar peduncle and part of the midbrain to the pons. CSF examination showed no increase in HAM/TSP biomarkers (neopterin 8 pmol/mL and CXCL10 796.0 pg/mL). Clonality analysis of HTLV-1-infected cells was performed using the rapid amplification of integration site method.^[12]^ Although monoclonal proliferation could be proven with DNA derived from the lymph node, the clonality of the CSF cells and peripheral blood mononuclear cells was polyclonal (Fig. 4). Since lymphadenopathy progressed, systemic chemotherapy was administered for lymphoma-type ATL. The cervical lymph nodes shrank, and the hyperintensity of the cervical cord to the central thoracic cord as seen on cervical thoracic spine MRI decreased after 2 courses of CHOP (cyclophosphamide, hydroxydaunorubicin, vincristine, and prednisone/prednisolone) without vincristine due to severe constipation. The soluble IL-2 receptor level also decreased from 1241 U/mL before CHOP therapy to 702 U/mL. However, despite 8 additional courses of mogamulizumab, the neurological symptoms did not improve.

## 3 Discussion and conclusions

The patient in the present case developed the clinical features of rapidly progressive HAM/TSP and was diagnosed with lymphoma-type ATL by lymph node biopsy. One month after onset, he was unable to stand because of spastic paraparesis, and after excluding other diseases by imaging examinations, the anti-HTLV-1 antibody titer was confirmed by CSF examination. This patient met the diagnostic criteria for rapidly progressive HAM/TSP.^[8]^ The biomarkers of disease activity, neopterin, and CXCL10, and the CSF inflammation level tended to be lower than that of typical rapidly progressive HAM/TSP.^[8]^ In the present case, abnormal lymphocytes were transiently seen in the CSF, but on clonality analysis of CSF cells, no ATL cells were detected.

A total of 5 cases of HAM/TSP associated with ATL were identified in the literature search. There were 3 cases of ATL after diagnosis of slowly progressive HAM/TSP (cases 1–3), rapidly progressive HAM/TSP-like symptoms caused by central nervous system (CNS) leukemia (case 4), and acute myelitis resembling HAM/TSP (case 5) (Table 2). The clinical course of cases 1 to 3 is considered to be slowly progressive HAM/TSP, and ATL developed about 20 years after the onset, which may have been a coincidence.^[13–15]^ Case 4 showed a rapidly progressive HAM/TSP-like clinical course with CSF testing negative for anti-HTLV-1 antibody but infiltration of ATL-like cells. This case was quite exceptional, suggesting that direct infiltration of ATL cells into the CNS caused neurological abnormalities.^[16]^ Case 5 presented with progressive paralysis 5 years after being diagnosed with chronic ATL. As in our case, the CSF test was positive for anti-HTLV-1 antibody, but no ATL cells were observed. The clinical diagnosis was considered to be rapidly progressive HAM/TSP rather than ATL with spinal cord infiltration. However, autopsy findings suggested that ATL cells infiltrated the spinal cord and elicited a local immune response. Therefore, it was suggested that chronic ATL was transformed into acute ATL, and infiltration of ATL cells into the spinal cord caused acute myelitis similar to HAM/TSP.^[17]^ Similar to case 5, ATL cells may have infiltrated the spinal cord and induced local immune responses in the present patient, whose clinical manifestations were similar to those of rapidly progressive HAM/TSP. Both the present case and case 5 had severe symptoms mimicking HAM/TSP, but the cases were completely different from HAM/TSP in that steroids or chemotherapy had no therapeutic effect. Therefore, further investigation is necessary to establish an appropriate treatment for neurological symptoms in ATL patients with suspected CNS infiltration.^[18]^

**Table 2:** Previous literature of HAM/TSP and HAM/TSP like symptoms with ATL.

Case no.	Age	Sex	Clinical course			Type of HAM/TSP	Steroid effect	Type of ATL	Author et al, year (citation)
1	46	F	HAM/TSP	→	ATL	Slow progressor	Yes	Lymphoma-type	Murata et al, 1990^[13]^
2	47	F	HAM/TSP	→	ATL	Slow progressor	Yes	Acute-type	Furukawa et al, 1995^[14]^
3	55	F	HAM/TSP	→	ATL	Slow progressor	Not listed	Acute-type	Takeda et al, 2020^[15]^
4	79	F	HAM/TSP like symptoms	→	ATL	Rapid progressor	No	Acute-type	Haruki et al, 2009^[16]^
5	55	M	ATL	→	HAM/TSP like symptoms	Rapid progressor	No	Chronic→acute-type	Wada et al, 2017^[17]^

ATL = adult T-cell leukemia/lymphoma, HAM/TSP = human T-cell leukemia virus type 1-associated myelopathy/tropical spastic paraparesis.

## Acknowledgments

The authors would like to acknowledge the support staff at the laboratory.

## Author contributions

All the authors stated above made substantive intellectual contributions to a published case report. KT treated the patient and wrote the manuscript. HM, ST, and SF treated the patient in the field of neurology. MG and YTa treated the patient in the field of hematology. TM analyzed the flow cytometric analysis of the CSF cells. YY helped in making the diagnosis of the patient by performing clonality analysis. YTs participated in the treatment of the patient and helped draft the manuscript. All authors read and approved the final manuscript.

**Conceptualization:** Yoshio Tsuboi, Yoshihisa Yamano.

**Data curation:** Yoshio Tsuboi, Keiko Tamaki, Hidekazu Mera, Sho Takeshita, Shinsuke Fujioka, Maki Goto, Taichi Matsumoto.

**Formal analysis:** Yasushi Takamatsu.

**Investigation:** Taichi Matsumoto, Yasushi Takamatsu.

**Supervision:** Yoshihisa Yamano.

**Writing – original draft:** Keiko Tamaki.

**Writing – review & editing:** Yoshio Tsuboi, Yoshihisa Yamano, Yasushi Takamatsu.
